# A Novel Platform for Evaluating the Environmental Impacts on Bacterial Cellulose Production

**DOI:** 10.1038/s41598-018-23701-y

**Published:** 2018-04-10

**Authors:** Anindya Basu, Sundaravadanam Vishnu Vadanan, Sierin Lim

**Affiliations:** 1 School of Chemical and Biomedical Engineering, Nanyang Technological University, 70 Nanyang Drive, Singapore, 637457 Singapore; 2 School of Pharmaceutical Sciences, Rajiv Gandhi Technical University, Bhopal, India

## Abstract

Bacterial cellulose (BC) is a biocompatible material with versatile applications. However, its large-scale production is challenged by the limited biological knowledge of the bacteria. The advent of synthetic biology has lead the way to the development of BC producing microbes as a novel chassis. Hence, investigation on optimal growth conditions for BC production and understanding of the fundamental biological processes are imperative. In this study, we report a novel analytical platform that can be used for studying the biology and optimizing growth conditions of cellulose producing bacteria. The platform is based on surface growth pattern of the organism and allows us to confirm that cellulose fibrils produced by the bacteria play a pivotal role towards their chemotaxis. The platform efficiently determines the impacts of different growth conditions on cellulose production and is translatable to static culture conditions. The analytical platform provides a means for fundamental biological studies of bacteria chemotaxis as well as systematic approach towards rational design and development of scalable bioprocessing strategies for industrial production of bacterial cellulose.

## Introduction

With increasing demands for novel materials with diversified applications, bacterial cellulose (BC) has been gaining attention within the scientific community over the past decades^1,2^. With the advent of synthetic biology, BC producing microbes are being developed as a novel chassis^3^. For this purpose, investigations on optimal growth conditions, impacts of carbon sources on the BC production, and understanding of the fundamental biological processes are paramount.

BC is devoid of hemicellulose and lignin, which are the common contaminants of plant cellulose, making it the purest forms of cellulose obtainable as-produced and is desirable for greener synthesis option. BC has found wide-spread applications in diversified fields particularly within the biomedical and electronics industries^2,4^. However, its large-scale production for such industries is challenged by the lack of productivity stemming from the limited biological knowledge of the corresponding bacterial strains. For example, bacterial growth is not always associated with cellulose production^5^. Under cellulose producing conditions, it is difficult to monitor the bacterial growth rates, thereby making it extremely challenging to test the associated cellulose productivities under varied conditions. To further understand the cellulose production, bacteria such as *Gluconacetobacter xylinus*, which spontaneously convert glucose into its polymeric form, cellulose, has become the model organism in this field^6^. A platform for quick screening of optimal cellulose producing conditions is particularly beneficial in circumventing practical difficulties associated with culturing of the bacteria by providing answers to some of these biological questions: Does the bacteria have preference towards carbon source and environmental conditions for growth or cellulose production? Can the bacteria perform chemotaxis and exhibit behaviour of common flagellate bacteria?

**Figure 1:**
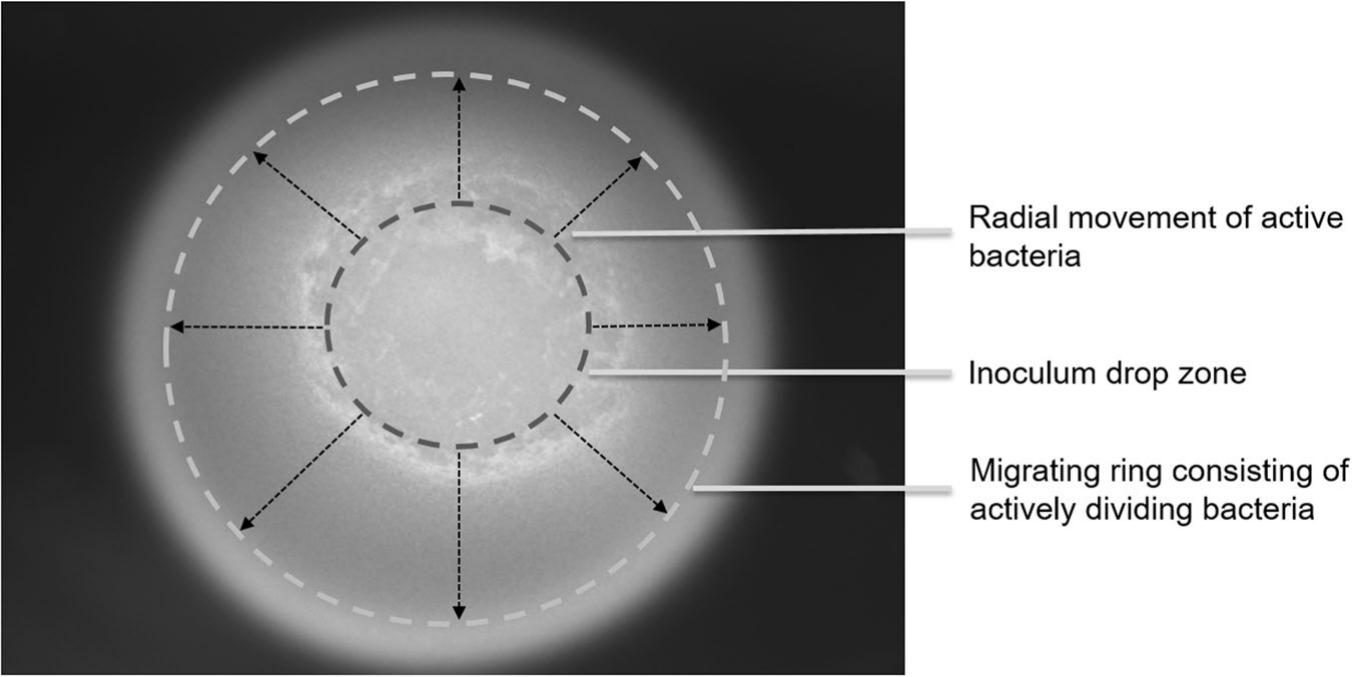
The expected growth pattern of a chemotactic bacteria on a soft agar swim plate set-up. The bacteria within the inoculum, when added to the central part of the plate, consume all carbohydrates at the region and form a ring of actively growing cells at the periphery. The chemotactic ring moves in all directions in search of fresh carbon source leaving behind dead or dying cells.

Leveraging on the preference of BC-producing bacteria to grow on substrate-surfaces^4^, owing to its aerobic nature, we hypothesize that surface growth models will shed deeper insights with regard to its biology^7^. Surface growth of chemotactic bacteria has been previously studied by several groups with Keller-Segel type as the widely accepted model^8,9^. In such experimental setup, a small drop of bacterial inoculum is added on top of a soft-agar medium surface at the centre of a petri-dish/plate and the bacteria is allowed to grow. Figure 1 and S1A (Supplementary Information) show the expected growth pattern. As the carbon source at the site of inoculation is consumed, the bacteria tend to form a concentric ring of actively growing cells which propagates in all directions simultaneously while leaving behind dead or dying cells^10,11^. Aligned to the same set-up, we establish the first screening platform by monitoring BC-producing bacteria surface growth under different conditions. We validate the applicability of the platform using three different strains that are *Gluconacetobacter hansenii* ATCC 53582, *Gluconacetobacter xylinus* ATCC 700178 and *Komagataeibacter rhaeticus* iGEM, under different growth conditions. We further establish that the trends obtained through the surface-growth platform correlate well with cellulose yields achievable under static culture conditions.

## Results

### All strains exhibit chemotaxis

**Figure 2:**
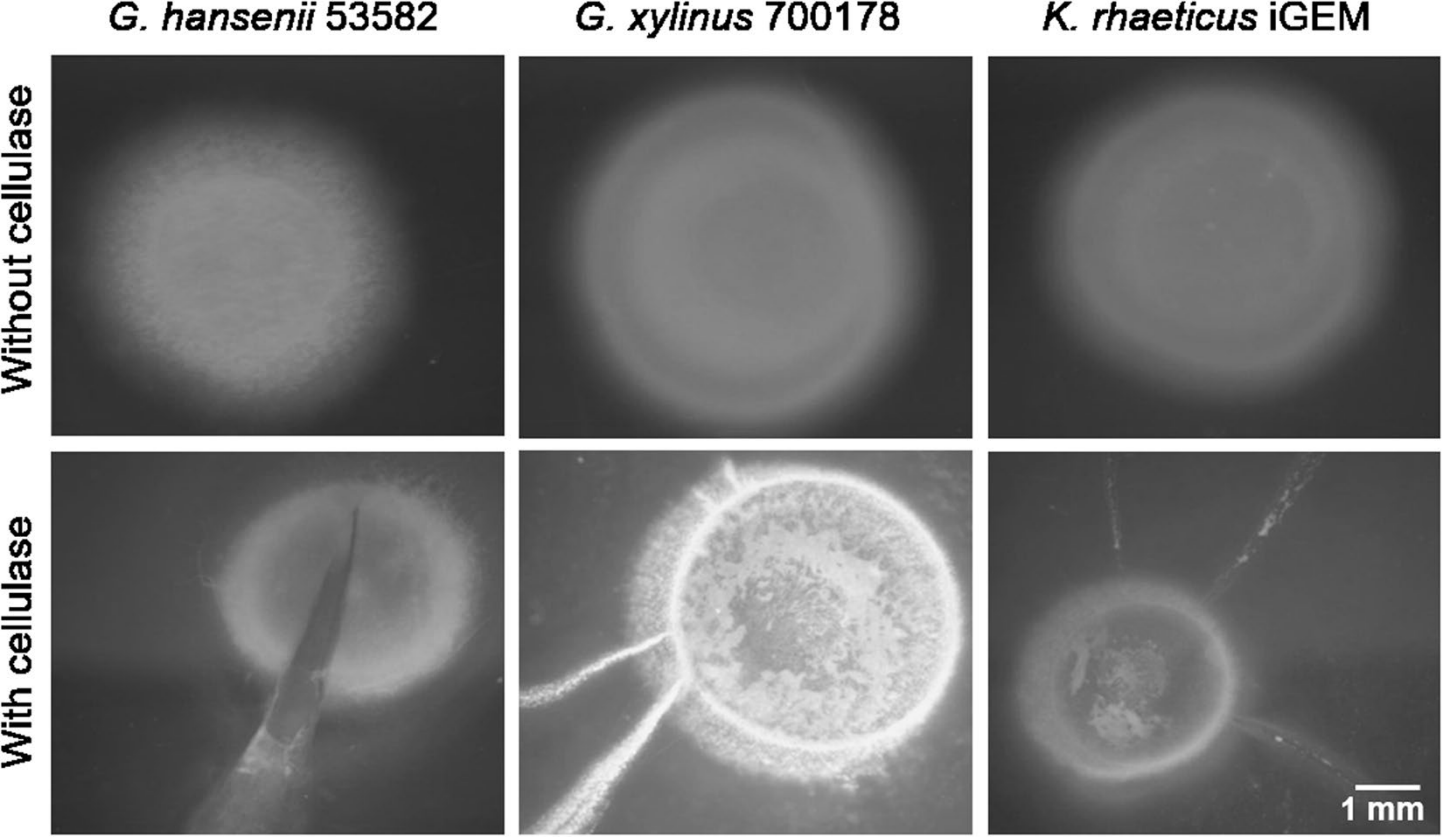
Swim plate experiments conducted for different cellulose producing bacterial strains in the absence (top panel) and the presence (bottom panel) of cellulase. The cultures were grown on soft agar in a humidified incubator.

**Figure 3:**
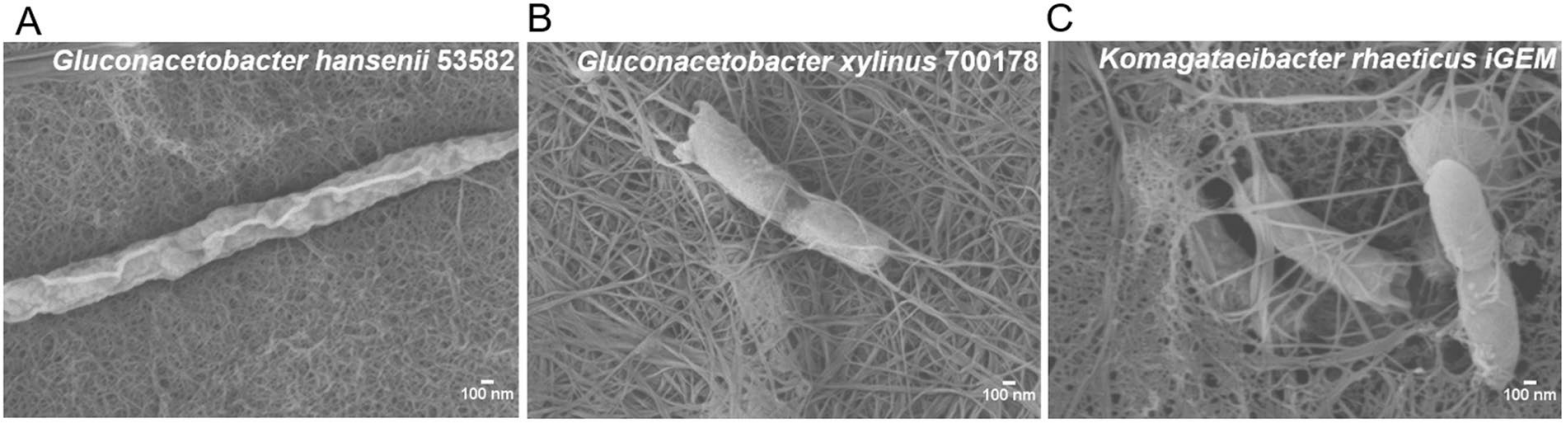
FESEM images of the pellicles grown on soft agar. (**A**) *Gluconacetobacter hansenii* 53582, (**B**) *Gluconacetobacter xylinus* 700178 and (**C**) *Komagataeibacter rhaeticus* iGEM; the pellicles were scooped out from the surface of the swim plates.

Upon incubation under a given condition, all strains produced visible BC pellicles within one day of incubation. At the initial phase, the pellicles resembled the shape and size of the inoculum droplet and subsequently grew into a concentric circle away from the drop zone as shown in Fig. 2 (top panel) and S1A. To examine their morphologies, the pellicles were carefully scooped out of the soft agar surface and visualised under a Field Emission Scanning Electron Microscope (FESEM). It was evident that for all the cases that the pellicles were being formed by fibres generated by our target organisms (Fig. 3).

Based on the previously discussed Keller-Segel surface growth model of bacteria, we expected that the increase in the bacterial population at the site of inoculation would lead to depletion of nutrients with time. Under such conditions, bacterial strains capable of exhibiting chemotaxis start moving towards the adjacent regions of higher nutrient concentration leaving behind the dead or dying cells^9,10^. To test this hypothesis on our strains, the pellicles were stained with a mixture of propidium iodide and Syto9 to determine the live/dead bacterial population distribution. Both dyes intercalate with DNA. Propidium iodide penetrates only dead bacterial cells resulting in red fluorescence while Syto9 stains live bacteria green^12–15^.

**Figure 4:**
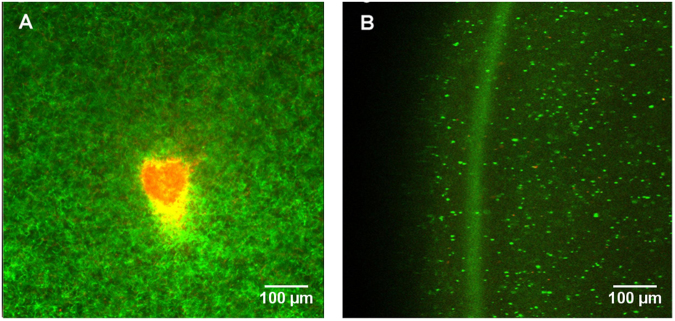
Typical spatial distribution of the live/dead bacteria within a cellulose pellicle grown on soft agar. Live bacteria are stained green while the dead ones are stained red. The centre of the pellicle (**A**) is marked by the presence of dead or dying cells while actively growing cells are seen at the edges of a pellicle (**B**).

Figure 4 presents a characteristic bacteria population profile at the centre and edges of the pellicles. The centre region of the pellicle is marked by a dense red coloured spot indicating the predominance of dead bacterial cells within the region. The red spot is surrounded by an orange-yellow coloured zone typically because of varying intensities of green and red fluorescence indicating the presence of dead or dying cells. However, towards the edges of the pellicle, we observe a green coloured ring (Fig. 4B) indicating that the region has high concentration of actively growing cells. The spatial distribution pattern of the bacterial population appears similar to that expected in the case of chemotactic bacteria exhibiting a Keller-Segel type growth^10^. Actively growing bacterial population keeps moving forward along the edges of the ring formed, leaving behind dead or dying cells particularly at the centre region where the inoculum was added. Based on our observations, we propose that the three strains in this study are capable of exhibiting chemotaxis and the pellicle formation could be an integral part of its chemotaxis. Although the involvement of BC with the bacterial movement has been previously observed by other research groups^16–18^, to the best of our knowledge this is the first observed direct correlation of the bacterial movement with chemotaxis.

### Cellulose production plays a role in chemotaxis and maintenance of bacterial metabolic state

To understand the importance of cellulose production to bacterial motility and its metabolic state, we grew the bacteria in the presence of cellulase in the soft agar medium. Cellulase is an enzyme known to degrade cellulose into smaller mono/oligosaccharide units^19^. We observed that the pellicles were formed on the first day but disintegrated by the second day. Subsequently, the bacteria grew in an unpredictable manner without following any concentric pattern distinct from the characteristic of bacteria showing chemotactic behaviour. The erratic growth corresponded to the disintegration of the cellulose fibres (Fig. 2, bottom panel and S1B). For genuine chemotactic bacteria, we expected the bacteria to retain its ring formation despite the loss of the cellulose fibres. The observed inconsistent behaviour of the three BC-producing bacteria compared to those incubated in the absence of cellulase are shown in Fig. 2 (top panel). Furthermore, under prolonged incubation in the presence of cellulase, the bacteria convert into non-cellulose producing metabolic state(s), where no pellicle formation is observed (data not shown). Our results indicate that under the experimental conditions, cellulose production might be an integral part of the bacterial chemotaxis. Consequently, in the absence of the fibres to support its movement, the bacteria probably switch to a different set of metabolic machineries to support its survival. The causes of the switch between cellulose producing and non-producing metabolic states are not yet established and remain to be elucidated.

### Bacterial strains show preference in carbohydrate utilization

As we have established that chemotaxis is associated with the production of cellulose fibres, we expect that a higher rate of chemotaxis correlates with an increase in cellulose production. We test the applicability of the surface growth model as an easy platform for determining the impact of diverse environmental conditions on the growth of the bacteria. Developing the platform will be useful for cost effective screening of the growth conditions necessary for maximal cellulose production. Briefly, the method quantifies growth effects by comparing the pellicle diameters in different experimental conditions. To test the platform, we first performed the swim plate experiments in the presence of four different carbon sources (i.e. glucose, fructose, sucrose and mannitol) at various concentrations. Glucose being one of the most widely used 6-carbon monosaccharide easily utilized by all bacteria; Fructose on the other hand (an isomer of glucose with a 5-membered furan ring) was chosen to evaluate the impact of the altering geometries of the carbohydrate molecule. Sucrose was used to understand the impact of a disaccharide, while mannitol (an open chain 6-carbon monosaccharide) was used to evaluate the impact of open chain carbon source on bacterial growth.

**Figure 5:**
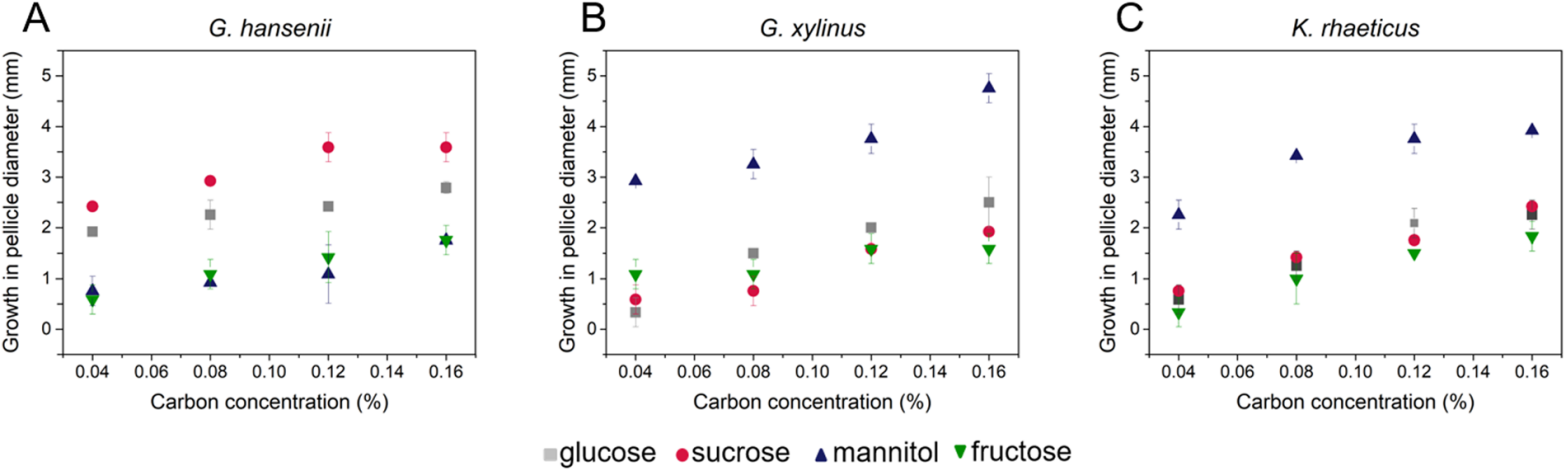
Observed growth of the different BC-producing strains grown under different carbon sources at different concentrations; (**A**) *Gluconacetobacter hansenii* 53582, (**B**) *Gluconacetobacter xylinus* 700178 and (**C**) *Komagataeibacter rhaeticus* iGEM.

**Figure 6:**
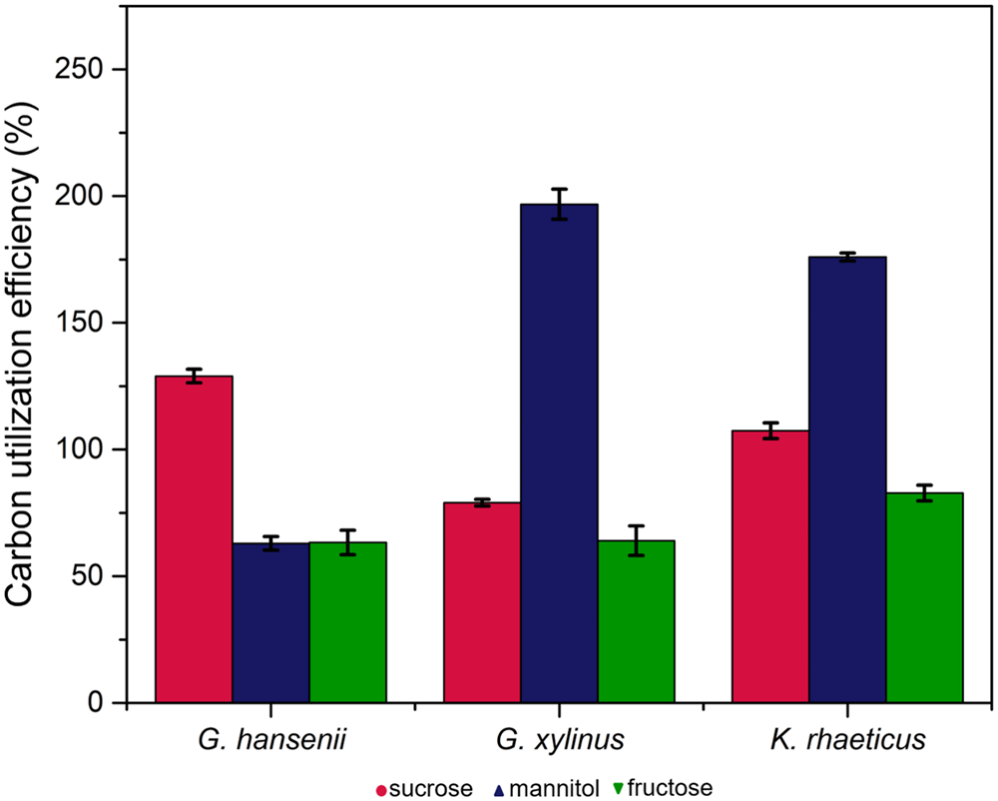
The impact of different carbon sources on the growth pattern of cellulose producing bacteria as derived from the swim plate experiments. The initial concentration of the carbon source is 0.16% (w/v). Carbon utilization efficiency for each of the strain is calculated with respect to glucose.

The observed pellicle growth profiles, shown in Fig. 5, present a few interesting points. First, the bacterial strains show obvious preferences towards the carbon sources used for their growth. *G. hansenii* 53582 strain showed preference towards sucrose with the order of preference towards other sources: Sucrose > Glucose > Fructose ≈ Mannitol. In contrast to *G. hansenii* 53582, both strains *G. xylinus* 700178 and *K. rhaeticus* iGEM show preference towards mannitol while their growth are unaffected by the other carbon sources. It is observed that the bacterial growth is proportional to sugar concentration (Fig. 5). However, for *G. hansenii* the growth reaches a plateau at 0.12% (w/v) sucrose. These results indicate that our swim plate experiments provide quantitative prediction for cellulose production capacity in different carbon sources. Figure 6 presents the observed advantage in cellulose production capacity at 0.16% (w/v) carbon source for different bacteria. The higher the observed area covered with cellulose produced by the bacteria grown on different carbon sources compared to glucose, the higher the growth rate and preference for the corresponding carbon source. Our platform thus allows quantification of the cellulose production between different sugars allowing to model bacterial growth under different scenarios.

### pH change often initiates significant changes in carbon utilization by the bacteria

**Figure 7:**
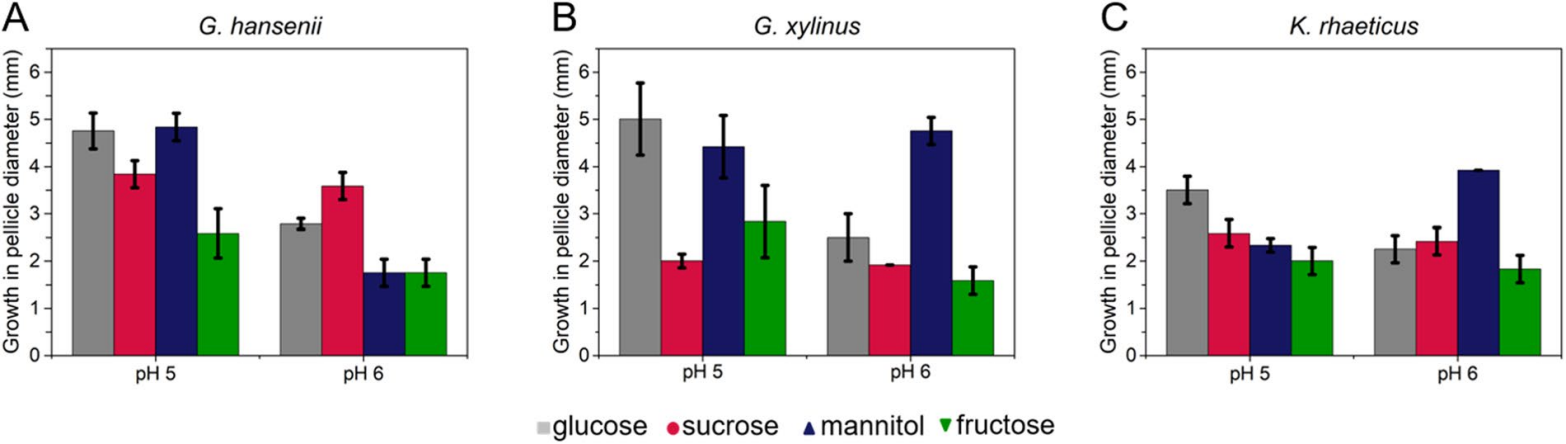
Testing the impact of pH on the growth or cellulose production capacity of the bacteria through the developed swim plate experimental platform. (**A**) *Gluconacetobacter hansenii* 53582, (**B**) *Gluconacetobacter xylinus* 700178 and (**C**) *Komagataeibacter rhaeticus* iGEM were grown on the different carbon sources at 0.16% (w/v).

To test its applicability in testing other growth influencing factors, we challenged our platform for evaluating the impact of pH. Pellicle formation was tested over a varied pH range of 2–8. The impact of pH drop on the bacterial growth profile is summarised in Fig. 7 where significant increase in bacterial growth is observed for all the strains grown on glucose at pH 5 compared to pH 6. Apart from glucose, *G. hansenii* exhibits significant increase in pellicle diameter for mannitol at pH 5 as well. Growth on sucrose and fructose supplemented media, on the other hand, barely affect *G. hansenii*. Interestingly, mannitol-fed *K. rhaeticus* shows a decrease in growth at pH 5 compared to that at pH 6. In addition, pellicle formation is only observed at pH 5 and 6, whereas other conditions show no signs of pellicle formation. This observation is in accordance to the growth patterns of the bacteria as observed by different research groups where cellulose production has been proposed to be optimum at 4 < pH < 7^1,4^. In general, the BC producing strains are known to grow under mild acidic conditions. However, the gluconic acid produced during subsequent cellulose production reduces the pH of the growth medium thereby making it difficult for further BC production^4,5^. These results therefore validate our platform for screening of BC producing conditions.

### Findings of the swim plate experiments translate well to static culture experiments

**Figure 8:**
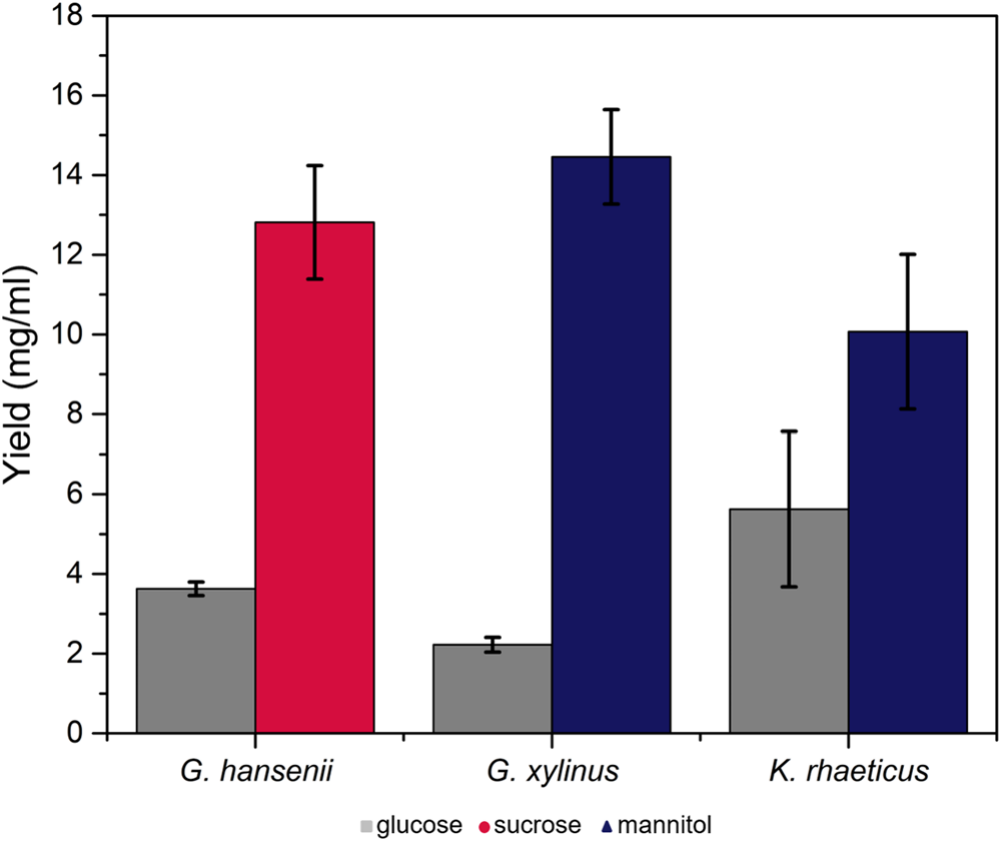
Comparison of the cellulose yields obtained using various static culture conditions. *G. hansenii* was grown in the presence of glucose and sucrose at 2% (w/v); *G. xylinus* and *K. rhaeticus* were grown in glucose and mannitol at 2% (w/v). The results show good correlation with the swim plate experiments.

For bioprocessing applications, it is necessary to extrapolate the observations on the platform to static cellulose production conditions. Identifying alternative carbon sources for desired bacterial growth conditions is important for improving yield. Our attention is particularly drawn to the fact that our bacteria show preferences for carbon sources other than glucose. *G. hansenii* was therefore grown in the presence of sucrose while *G. xylinus* and *K. rhaeticus* were grown in the presence of mannitol. For validation purposes, the yields obtained from the above carbon sources are compared to that obtained from glucose (Fig. 8). Cellulose yields under static conditions agree well with the predictions obtained from our swim plate experiments where *G. hansenii* preferred sucrose over glucose, while mannitol is found to be the preferred carbon source for *G. xylinus* and *K. rhaeticus*.

## Discussions

The overall goal of the present work is to establish a simple screening platform for BC production which can simultaneously shed new light on the biology of the bacteria. Our particular interest is to compare the growth pattern with the Keller-Segel type growth primarily due to its compatibility with further mathematical modelling^20^. Preliminary results presented here (Figs 2 and 4), suggest that the surface-growth pattern of the three bacterial strains in this study resemble Keller-Segel models consisting of actively growing cells at the edges of the pellicles leaving behind dead or dying cells in the central region^10^. Researchers working with bacterial cellulose have two distinct school of thoughts regarding the *G. xylinus* motility: (1) flagellate movement and (2) propulsion facilitated by inverse ejection force of the produced cellulose fibres^16,18^. Observations using our platform confirms the second school of thoughts. While others have reported cellulose-mediated motility without relating it to chemotaxis, we note an interesting correlation. In our experiments, we find that cellulose fibres are produced by the bacteria within the pellicles (Fig. 3) and the organism deviates from its conventional chemotactic behaviour when incubated in presence of cellulase (Fig. 2). Consequently, the observations bring up an obvious question mark on the extent to which the bacteria adhere to the surface growth patterns observed in case of flagellate propulsion. A closer look into our experimental data reveals obvious differences between the movement of the three strains and flagellate movement. In the latter case, the velocity of the bacterial front remains independent of the sugar concentration within the growth media^10^ which is the opposite of what we observed. The similar observations for all three strains imply that the chemotactic behaviour would be universal for cellulose mediated bacterial propulsion systems. Nevertheless, the BC producing strain growth remains associated with another interesting observation. On some occasions, the bacterial front achieves a terminal velocity characterized by a plateau in pellicle diameter despite the increase in sugar content (Fig. 5A) suggesting a plausible limit of the extent of resources that can be allocated by the bacteria for exhibiting chemotaxis. Our experimental swim-plate platform indicates that obvious relationships do exist between pellicle diameter and sugar concentration, which can be used to derive empirical or semi-empirical growth models.

The good correlations between the observed trends in our swim-plate platform and that obtained in static cultures confirm that the platform developed to understand biological questions is translatable to predictive models for bioreactor studies for these bacteria. Our proposed platform can be particularly useful to address some of the practical difficulties whilst studying the bacterial growth profile within bioreactors. For instance, the pH of the media plays a vital role in determining the cellulose productivities of the target organisms. However, owing to the development of cellulosic coats on the surface of the pH probe, it is difficult to monitor the dynamic changes of the medium pH during fermentation. In the present experimental platform, such problems can be easily eliminated since the pH of the media remains predetermined as the bacteria are moving towards fresh resources.

We therefore propose that the current experimental platform can be used as a quick screening methodology for determining the optimal growth conditions in addition to better understanding the biology of the bacteria. The combination of the biological knowledge and the platform has implications on the establishment of optimised bioprocessing strategies for BC large-scale production.

## Methods and Materials

All chemicals were purchased from Sigma Aldrich, unless specified otherwise.

### Culture method and organisms

Three cellulose producing strains were considered for this study, *Gluconacetobacter hansenii* ATCC 53582, *Gluconacetobacter xylinus* ATCC 700178 and *Komagataeibacter rhaeticus* iGEM (a kind gift from Prof. Tom Ellis, Imperial College, London) were grown in Hestrin-Schramm (HS) medium. Carbon sources such as glucose, sucrose, fructose and mannitol were substituted into the culture medium according to the experimental study design. A pre-inoculum was prepared in petri-plates in HS media with 2% (w/v) of the desired sugar and allowed to grow for at least 2–3 days under static conditions at 26 °C. The BC pellicles formed were removed and the cell suspensions present in the dish were used as inoculum for the experimental studies.

### Swim plate experiments

The inoculum (OD_600_ = 0.005 to 0.01) at 10 µl was carefully dropped at the centre of each well of six-well or twelve-well culture plates containing HS-agar medium in the presence or absence of cellulase (obtained from *Trichoderma reesei*, ATCC 26921). Agar concentration within the HS medium was maintained at 0.15% since higher agar concentrations lead to increased viscous drag thereby limiting bacterial movement. Lower agar concentrations were attempted but were rendered difficult to handle in addition to minimal improvement to the experimental set-up. The culture suspensions were allowed to grow in a well-humidified incubator under static conditions at 26 °C. The growth pattern of the surface pellicles was monitored using a light microscope with a 5 x objective lens. The diameter of the pellicles, determined as the largest end-to-end distance, was used to compare the bacterial growth conditions. Each of the three independent experiment was conducted in triplicates and the data obtained were represented as mean ± standard error.

### Morphology observations

#### Field emission scanning electron microscopy

The pellicles formed on the surface of the agar plates were carefully scooped out and washed with phosphate buffered saline (PBS, 1x). The samples were kept on glass cover-slips and dried using graded concentration series of ethanol prior to visualization under a Field Emission Scanning Electron Microscope (JSM-6700F, JEOL, Japan) using standard protocols^15,21^.

#### Confocal microscopy

Pellicles formed on the swim plates were carefully scooped out from the surface and washed at least thrice in PBS. The samples were then stained with the Baclight LIVE/DEAD assay kit (Life Technologies) as described elsewhere^13^ along with minor modifications. Briefly, equimolar quantities of the dyes: Syto9 and Propidium iodide, were mixed together and diluted ten times in 1% NaCl solution. The washed pellicles were added into the dye solution and allowed to incubate for 10 minutes in the dark. The pellicles were then immediately visualised under a confocal microscope.

### Static culture experiments

Static cultures simulating bioreactor conditions for cellulose production were used for growing the desired strains under various conditions at 26 °C. Culture volumes ranging from 2 ml to 150 ml were grown in vessels of varied dimensions: starting from 12-well culture plates to 135 mm petri-dishes. Glucose or sucrose at media-concentrations ranging from 2% to 8% were used as the carbon source for *G. hansenii*, while *G. xylinus* and *K. rhaeticus* were grown in the presence of 2% to 4% glucose or mannitol. The bacterial cellulose pellicles obtained were treated in 0.5 M NaOH at 100 °C for 10 minutes to eliminate the entrapped cells and washed with deionized water until the pH was neutral. The pellicles were subsequently freeze dried and weighted using a high precision balance. The yield was defined as the mass of the dried cellulose pellicle obtained per mL of culture. Carbon utilization efficiency was defined as the ratio of pellicle diameter for the preferred carbon source to the pellicle diameter of cellulose obtained from glucose as the carbon source. All experiments were done in triplicates and the data obtained were represented as mean ± standard error.

## Electronic supplementary material

Supplementary Information
