# Supplementary material for: A Novel Platform for Evaluating the Environmental Impacts on Bacterial Cellulose Production
Source: Sci Rep. 2018 Apr 10;8:5780. doi: 10.1038/s41598-018-23701-y (PMC5893554; doi:10.1038/s41598-018-23701-y)
Supplement: Supplementary file 1 — Supplementary Information [file 41598_2018_23701_MOESM1_ESM.pdf]

A Novel Platform for Evaluating the Environmental Impact on Bacterial Cellulose Production

Anindya Basu[a,b], Sundaravadanam Vishnu Vadanan[a], Sierin Lim[a]*

[a] School of Chemical and Biomedical Engineering, Nanyang Technological University, Singapore

[b] School of Pharmaceutical Sciences, Rajiv Gandhi Technical University, Bhopal, India

* Corresponding Author:

Sierin Lim

Nanyang Technological University

School of Chemical and Biomedical Engineering

70 Nanyang Drive

Singapore 637457

Office: Block N1.3-B3-11

Supplementary Information

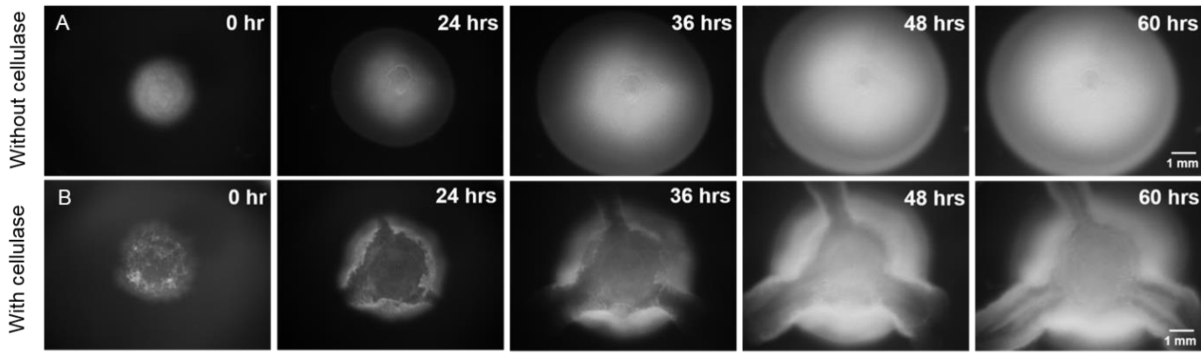

Figure S1 Typical growth patterns of *G. xylinus* 700178 bacteria grown in the absence (A) and in the presence (B) of cellulase at different time points during incubation.
